# Hepatic embolization for Cushing syndrome from metastatic tumors: a single-center case series

**DOI:** 10.1210/jendso/bvag046

**Published:** 2026-02-26

**Authors:** Shuyao Zhang, Jorge Esteban Mosquera, Clive Musonza, Anil K Pillai, Jessica Abramowitz, Sasan Mirfakhraee, Patricio M Polanco, Salwan Al Mutar, Waddah Arafat, Oksana Hamidi

**Affiliations:** Department of Internal Medicine, Division of Endocrinology, University of Texas Southwestern Medical Center, Dallas, TX 75390, USA; Department of Internal Medicine, Division of Endocrinology, University of Texas Southwestern Medical Center, Dallas, TX 75390, USA; Department of Radiology, Division of Vascular & Interventional Radiology, University of Texas Southwestern Medical Center, Dallas, TX 75390, USA; Department of Radiology, Division of Vascular & Interventional Radiology, University of Texas Southwestern Medical Center, Dallas, TX 75390, USA; Department of Internal Medicine, Division of Endocrinology, University of Texas Southwestern Medical Center, Dallas, TX 75390, USA; Department of Internal Medicine, Division of Endocrinology, University of Texas Southwestern Medical Center, Dallas, TX 75390, USA; Department of Surgery, Division of Surgical Oncology, University of Texas Southwestern Medical Center, Dallas, TX 75390, USA; Department of Internal Medicine, Division of Hematology and Oncology, University of Texas Southwestern Medical Center, Dallas, TX 75390, USA; Department of Internal Medicine, Division of Hematology and Oncology, University of Texas Southwestern Medical Center, Dallas, TX 75390, USA; Department of Internal Medicine, Division of Endocrinology, University of Texas Southwestern Medical Center, Dallas, TX 75390, USA

**Keywords:** Cushing syndrome, hepatic embolization, yttrium-90 radioembolization, transarterial embolization, adrenocortical carcinoma, neuroendocrine tumor

## Abstract

**Background:**

Cushing syndrome (CS) from metastatic adrenocortical carcinoma (ACC) or neuroendocrine tumors (NETs) presents a therapeutic challenge when surgery is not feasible. Liver-directed embolization, including bland transarterial embolization (TAE) and yttrium-90 (Y-90) radioembolization, may palliate hypercortisolism arising from hormonally active hepatic metastases.

**Methods:**

We conducted a retrospective single-center case series of 4 adult patients (≥18 years) with CS and liver-dominant metastatic ACC or NET who underwent hepatic embolization between 2015 and 2025. Inclusion criteria were: (1) confirmed CS based on standard biochemical testing, and (2) receipt of liver-directed embolization (TAE or Y-90) for hypercortisolism. Exclusion criteria were absence of pre- and postembolization hormonal data preventing biochemical assessment. The primary outcome was biochemical response within 14 days (≥50% reduction or normalization of morning cortisol). Secondary outcomes included duration of biochemical control, radiographic response (RECIST 1.1), and adverse events (CTCAE v5.0).

**Results:**

Four patients (2 ACC, 2 NET) underwent Y-90 (*n* = 1) or TAE (*n* = 3). All achieved significant cortisol; 2/4 normalized cortisol with transient adrenal insufficiency. The Y-90 patient had sustained remission, while 2 TAE patients achieved partial hormonal control enabling tapering of medical therapy. Radiographically, tumor burden stabilized or improved in most treated lesions. Embolization was well tolerated, with only 1 case of post-embolization syndrome and no procedure-related mortality. One patient died from disease progression; 3 remain alive with controlled or improving disease.

**Conclusion:**

Hepatic embolization is a viable palliative option for CS due to unresectable liver-dominant metastases, providing meaningful biochemical improvement with acceptable safety and supporting integration into multidisciplinary CS management.

Cushing syndrome (CS) caused by ectopic adrenocorticotropin hormone (ACTH) secreting neuroendocrine tumors (NETs) or cortisol-secreting adrenocortical carcinoma (ACC) is a rare but clinically challenging condition, often presenting with severe hypercortisolism and limited treatment options [1, 2]. Treatment goals for patients with CS include tumor control and management of clinical symptoms and impaired quality of life by means of controlling excess cortisol production [3, 4]. Surgical resection is the preferred approach for localized disease. However, some patients present with unresectable metastases or are unsuitable for surgery [2, 5], where outcomes are unfavorable and medical therapy often fails to adequately achieve hormonal control [6, 7].

Because functional liver-dominant metastases can be the predominant source of cortisol or ACTH production, hepatic transcatheter arterial embolization (TAE) and yttrium-90 (Y-90) radioembolization have emerged as minimally invasive strategies that may reduce tumor perfusion and rapidly improve hormonal control [8]. The rationale for using hepatic arterial embolization-directed techniques lies in the liver's unique vascular anatomy. The liver receives ∼75% of its blood supply from the portal vein, while malignant liver tumors derive the majority (∼90-100%) of their blood supply from the hepatic artery. This differential allows for selective delivery of particles into the hepatic artery, where they are preferentially trapped in the hypervascular tumor bed while the normal hepatic parenchyma remains protected by its portal venous supply. Tumor-directed ischemia (TAE) or radiation-induced necrosis (Y-90) can therefore provide local tumor control and reduction in tumor burden independent of hormonal status [6, 7]. While these techniques are well-established in hepatocellular carcinoma and metastatic colorectal cancer [9, 10], their role in hormonally active tumors, such as those causing CS, remains insufficiently investigated.

We present 4 patients with CS where liver metastases were treated with hepatic embolization for palliative management. This series evaluates the biochemical and radiographic outcomes of embolization and reviews the current literature on its use in CS.

## Materials and methods

We conducted a single-center retrospective case series between September 1, 2015 and July 31, 2025 to evaluate the outcomes of liver-directed arterial embolization in patients with CS due to metastatic liver tumors. Only adult patients (age ≥18 years) were eligible for inclusion in this study. An Institutional Review Board approval was waived, and electronic health records were reviewed to identify patients who underwent hepatic embolization for ACC or NET. Our objective was to evaluate biochemical, radiographic, and safety outcomes.

Inclusion criteria were: (1) a biochemically confirmed diagnosis of CS [morning cortisol >1.8 µg/dL (50 nmol/L) following overnight 1 mg dexamethasone suppression test, elevated 24-hour urinary free cortisol (UFC), and/or elevated late-night salivary cortisol (LNSC) with ACTH concordance], and (2) liver-directed embolization (Y-90 radioembolization or bland TAE) performed specifically for controlling hypercortisolism. Exclusion criteria included absence of documented hormonal evaluation before and after embolization, which precluded assessment of biochemical response.

A total of 114 cases of liver-directed embolization for hormonally active liver metastases were evaluated at our center. Of these, 4 cases (3.5%) met all eligibility criteria for biochemically confirmed CS with liver-dominant metastatic disease and were included in this case series. The age range of the included patients was 18 to 63 years, with 18 years being the youngest adult participant. We extracted demographics, tumor characteristics, hormonal profiles [UFC, serum cortisol, ACTH, dehydroepiandrosterone sulfate (DHEA-S)], embolization technique parameters, and clinical outcomes. Imaging was assessed by RECEIST 1.1 when available. Laboratory results were reviewed to confirm diagnosis and assess treatment response.

## Clinical cases

The following 4 cases, summarized in Table 1, illustrate the clinical and biochemical outcomes of hepatic embolization in patients with CS. Embolization techniques varied—1 patient received Y-90 radioembolization, while the remaining 3 underwent bland embolization.

**Table 1:** Summary of clinical Cases

Case	1	2	3	4
Age/sex	40/M	49/F	63/M	18/F
Diagnosis	Metastatic ACC	Metastatic NET	Metastatic NET	Metastatic ACC
Peak serum cortisol µg/dL (nmol/L) Ref: <23 µg/dL, <634.8 nmol/L	49.6 (1369 nmol/L)	71.4 (1971.4 nmol/L)	59 (1628.4 nmol/L)	25.8 (712.1 nmol/L)
Nadir serum cortisol µg/dL (nmol/L)	6.9 (190.4 nmol/L)	13.8 (380.9 nmol/L)	2.2 (60.7 nmol/L)	8.3 (229.1 nmol/L)
ACTH pg/mL (pmol/L) Ref: 10 to 60 pg/mL, 2.2 to 13.2 pmol/L	<5 (1.1 pmol/L)	190 (41.8 pmol/L)	158 (34.8 pmol/L)	<5 (1.1 pmol/L)
Peak UFC µg/24hours (nmol/24 hours) Ref: <45 µg/24 hours, <124.2 nmol/24hours	209 (577 nmol/24 hours)	N/A	N/A	1022 (2821 nmol/24 hours)
Tumor type	Adrenocortical carcinoma	Well-differentiated NET	Well-differentiated NET	Adrenocortical carcinoma
Size of initial hepatic metastasis	8.7 cm	16 cm	14.1 cm	12.4 cm
Embolization type	Y-90 radioembolization (2 rounds)	Bland embolization (2 rounds)	Bland embolization	Bland embolization
Systemic therapy prior to embolization	Osilodrostat	Metyrapone, Osilodrostat		Osilodrostat
Follow-up time, months	32	24	1	3
Outcome	Biochemical remission; resumed mitotane with hydrocortisone	Biochemical remission; on hydrocortisone for adrenal insufficiency	Deceased; transitioned to hospice and passed away 33 days postembolization	Stable disease; on mitotane with hydrocortisone and osilodrostat

Abbreviations: ACC, adrenocortical carcinoma; ACTH, adrenocorticotropic hormone; NET, neuroendocrine tumor; UFC, urinary free cortisol.

### Case 1

A 40-year-old man with severe CS secondary to metastatic ACC presented with large, unresectable hepatic metastases. He was initially diagnosed with ACTH-independent CS after identification of a 6.9 cm heterogeneous right adrenal mass. Robotic laparoscopic adrenalectomy was performed at an outside institution, and pathology revealed an oncocytic neoplasm of uncertain malignant potential. He was treated with prednisone 5 mg daily for 4 months, which was subsequently discontinued.

**Figure 1:**
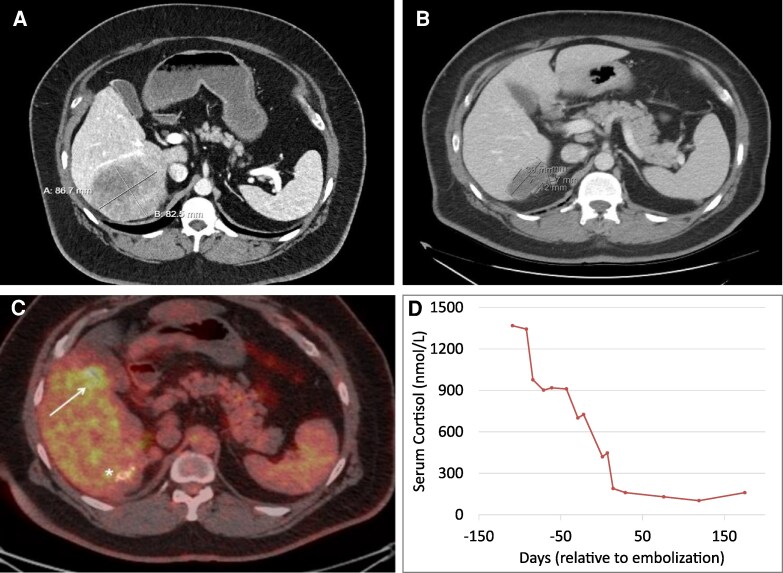
Response to hepatic embolization in case 1. Axial contrast-enhanced computed tomography images demonstrating a large centrally located hepatic metastasis prior to (A) and 7 months after (B) yttrium-90 embolization. The mass measured 8.7 × 8.3 cm pretreatment (A), with a marked reduction to 3.0 × 1.2 cm post-treatment (B). A follow-up 18F-FDG PET/CT (C) showed postradioembolization changes in segment 6 (*) and a new 4.4 cm hypermetabolic lesion in segment 5 near the gallbladder fossa (white arrow), along with new suprarenal nodules. Longitudinal serum cortisol trajectory following hepatic embolization procedure demonstrating sustained biochemical response following Y-90 embolization in Case 1 (D).

Approximately 1 year following adrenalectomy, the patient developed worsening hyperglycemia accompanied by recurrent Cushingoid features. Two years postoperatively, he presented to our institution with clinical evidence of recurrent CS. Biochemical evaluation revealed elevated 24-UFC of 209 µg/24 hours, reference <45 µg/24 hours (576.84 nmol/24 hours, reference <124.2 nmol/24 hours), LNSC of 0.95 and 0.63 µg/dL, reference <0.1 µg/dL (26.22 nmol/L and 17.39 nmol/L, reference <2.76 nmol/L), and suppressed serum ACTH <5 pg/mL, reference 10 to 60 pg/mL (<1.1 pmol/L, reference 2.2-13.2 pmol/L). Computed tomography (CT) demonstrated an 8.7 cm hepatic lesion in segment 6 with extension into segment 7 (Fig. 1A). 18-F Fluorodeoxyglucose positron emission tomography/CT (18F-FDG PET/CT) showed no evidence of extrahepatic disease.

Despite escalating osilodrostat dose to 6 mg twice daily, hypercortisolism remained uncontrolled. Surgical resection was attempted but aborted intraoperatively due to extensive peritoneal carcinomatosis, highlighting the limited sensitivity of PET imaging for detecting small-volume or miliary peritoneal disease. The patient subsequently underwent Y-90 radioembolization with TheraSpheres Y-90 glass microsphere (Boston Scientific). A total of 159 mCi was delivered to the segmental artery supplying the dominant hepatic lesion.

Fourteen days post-embolization, morning serum cortisol decreased from a peak of 49.6 µg/dL, reference: 6 to 23 µg/dL (1369 nmol/L, reference: 165.6-634.8 nmol/L) to 6.9 µg/dL (190.4 nmol/L), prompting initiation of hydrocortisone for adrenal insufficiency. Post-embolization CT scan showed a marked reduction of the lesion to 3.0 cm (Fig. 1B). Systemic chemotherapy with cisplatin, doxorubicin, and etoposide was initiated but discontinued after the first cycle due to septic shock and acute kidney injury requiring dialysis. Mitotane was trialed but the patient could not tolerate it due to gastrointestinal side effects. The patient was lost to follow-up for nearly 1 year due to loss of insurance.

Upon re-presentation, he reported recurrent Cushingoid symptoms, worsening diabetes, and hypertension. Morning cortisol was 19.6 µg/dL (540.96 nmol/L), ACTH remained suppressed <5 pg/mL (<1.1 pmol/L), and UFC was within normal limits at 37.9 µg/24 hours (104.7 nmol/24 hours). Repeat 18F-FDG PET/CT showed post-radioembolization changes in segment 6 and a new 4.4 cm hypermetabolic lesion in segment 5 near the gallbladder fossa (Fig. 1C), along with new suprarenal nodules. The previously treated segment VI liver lesion demonstrated interval decrease in surrounding FDG activity in keeping with resolving posttreatment change. A second Y-90 treatment was administered. Post-procedure labs showed UFC 31.9 µg/24 hours (88.0 nmol/24 hours), morning cortisol 11.5 µg/dL (317.4 nmol/L), and ACTH 7 pg/mL (1.54 pmol/L) (Fig. 1D).

At last follow-up, ∼32 months after initial Y-90 embolization, the patient remained in biochemical remission. Diabetes was well-controlled with tirzepatide and insulin. He is currently reattempting mitotane monotherapy with hydrocortisone replacement.

### Case 2

**Figure 2:**
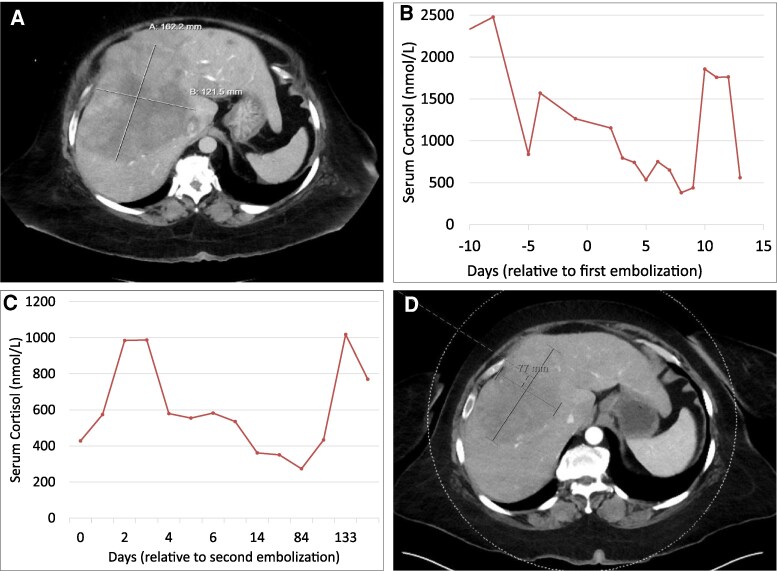
Radiographic response to bland embolization in case 2. Axial contrast-enhanced computed tomography image demonstrating a heterogeneous hepatic metastasis in segment 6 prior to bland embolization (A). The lesion measured 16.2 × 13.3 × 12.2 cm at baseline. Serum cortisol levels obtained around the time of the first bland embolization (B). Serum cortisol levels following the second bland embolization, illustrating a partial and delayed biochemical response (C). Axial contrast-enhanced computed tomography image 7 months after bland embolization, showing interval reduction of the hepatic lesion to 3.0 × 7.7 cm in Case 2 (D).

A 49-year-old woman presented with bilateral lower extremity edema, weight gain of 50 pounds (22.7 kg) over 6 months, and severe hypokalemia (serum potassium 2.8 mmol/L; reference: 3.5-5.1 mmol/L). Laboratory evaluation revealed ACTH-dependent CS with markedly elevated serum cortisol at 71.4 µg/dL (1970.6 nmol/L), plasma ACTH 190 pg/mL (41.8 pmol/L), and DHEA-S at 462 µg/dL, reference: 35 to 430 µg/dL (12.5 µmol/L; reference: 1.0-11.8 µmol/L). Computed tomography of the chest, abdomen, and pelvis demonstrated a 3.4 cm left adrenal nodule and multiple hepatic lesions, the largest measuring 16 cm (Fig. 2A). Given the presence of an adrenal nodule and severe hypokalemia, primary aldosteronism was evaluated. Aldosterone was suppressed <4.0 ng/dL (<140 pmol/L) with nonsuppressed renin of 1.0 ng/mL/hour (1 mcg/L/hour), arguing against autonomous aldosterone secretion. Liver biopsy revealed a grade 2 (G2) well-differentiated NET of unknown primary origin (<1 mitosis per 10 high-power fields, no necrosis, and Ki-67 index of 5-8%).

She was initiated on subcutaneous octreotide, spironolactone, and metyrapone. Despite uptitration of metyrapone to 3000 mg/day in divided doses, hypercortisolism remained poorly controlled. Due to extensive tumor burden and poor functional status, she was not a candidate for bilateral adrenalectomy or hepatic resection. She subsequently underwent 2 sessions of bland embolization targeting the dominant hepatic lesions.

Following the first embolization, serum cortisol declined from 63.9 µg/dL (1762.4 nmol/L) to a nadir of 13.8 µg/dL (381.7 nmol/L) by day 8 but rebounded to 67.3 µg/dL (1856.9 nmol/L) by day 10 (Fig. 2B). After the second embolization, cortisol decreased to 20.8 µg/dL (573.9 nmol/L) on day 1 but rose again to 35.7 µg/dL (986.0 nmol/L) by day 2 (Fig. 2C). Metyrapone was resumed at 500 mg 3 times daily, followed by a transition to osilodrostat to improve hormonal control and reduce pill burden. 18F-FDG PET/CT showed persistent hepatic metastases with decreased uptake in the most avid lesion and no new extrahepatic disease at 4 months postembolization (Fig. 2D). Ectopic CS remained well-controlled on osilodrostat 1 mg twice daily, which was later discontinued due to adrenal insufficiency. She is maintained on hydrocortisone replacement.

For management of metastatic NET, she completed 3 of 9 planned cycles of nivolumab and ipilimumab. A follow-up 18F-FDG PET/CT demonstrated increased uptake in several hepatic lesions concerning for progression, and she has since transitioned to FOLFOX chemotherapy. Over the course of follow-up, the adrenal lesion demonstrated radiographic behavior consistent with metastatic disease rather than a primary adrenal tumor. Despite disease progression, she remains active and has returned to full-time work. At last follow-up approximately 24 months after initial bland embolization, she has lost over 100 pounds (45.4 kg), returning to her baseline weight of 164 pounds (74.4 kg). Hypertension is in remission off all antihypertensive medications, and hypokalemia has resolved without supplementation.

### Case 3

**Figure 3:**
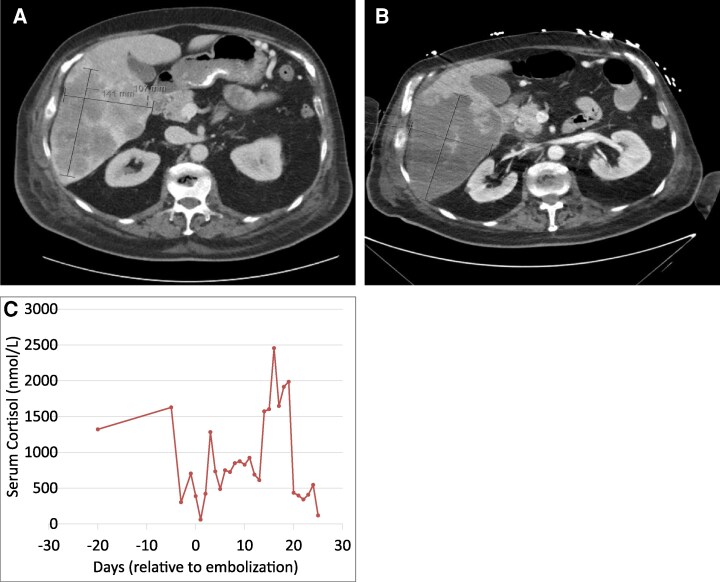
Short-term imaging follow-up after bland embolization in case 3. Axial contrast-enhanced computed tomography images demonstrating a dominant heterogeneous hepatic mass in the right hepatic lobe prior to (A) and 2 weeks after (B) bland embolization. The lesion measured 14.1 × 10.7 cm pretreatment (A) and 14.9 × 10.0 cm on early follow-up imaging (B), without significant size reduction. Minimal biochemical response observed following bland hepatic embolization in Case 3 (C).

A 63-year-old man presented with worsening back pain. Computed tomography of the abdomen and pelvis revealed a 2.8 cm mildly enhancing mass at the pancreatic body/tail junction with peripheral calcifications, innumerable centrally hypoattenuating liver lesions (largest measuring 14.1 cm in the right hepatic lobe; Fig. 3A), and spinal metastases. Liver biopsy confirmed metastatic, well-differentiated, grade 2 NET (Ki67 4.6%), immunohistochemically positive for AE1/AE3, synaptophysin, and chromogranin, and negative for Glypican-3, CK7, and CK20.

Biochemical evaluation was consistent with carcinoid syndrome: 24-hour urinary 5-hydroxyindoleacetic acid was elevated to 357 mg/24 hours, reference: ≤10.0 mg/24 hours (1867µmol/24 hours, reference: ≤52.3 µmol/24 hours), serum serotonin was 1010 ng/mL, reference: ≤330 ng/mL (5.73 nmol/L, reference: ≤1.87 nmol/L), and chromogranin A was 2380 ng/mL, reference: <93 ng/mL (0.049 nmol/L, reference: <0.002). The patient was started on continuous octreotide infusion.

Further workup revealed ACTH-dependent CS with random serum cortisol of 33 µg/dL (911.5 nmol/L) and ACTH of 158 pg/mL (34.76 pmol/L). Medical therapy was initiated with ketoconazole 200 mg every 8 hours and metyrapone 1500 mg daily in divided doses. Bilateral adrenalectomy was considered but deemed unfeasible due to poor surgical candidacy.

The patient underwent bland embolization of right hepatic lobe metastases using Embozene microspheres in a stepwise fashion: 100 µm (3 vials), 250 µm (2 vials), and 400 µm (2 vials), until stasis was achieved in the right hepatic artery.

On postembolization day 1, serum cortisol decreased from a peak of 59.0 µg/dL (1629 nmol/L) to 2.2 µg/dL (60.7 nmol/L) (Fig. 3B). Ketoconazole and metyrapone were held, and hydrocortisone was initiated for adrenal insufficiency. Cortisol levels began to rise, and metyrapone was restarted on day 4. Due to poor oral tolerance, osilodrostat 5 mg twice daily was administered via nasogastric tube.

The hospital course was complicated by grade 4 hypoxemia requiring mechanical ventilation, grade 4 hypotension requiring vasopressor support and opportunistic Pneumocystis jirovecii pneumonia. Postembolization CT showed necrotic changes in right hepatic metastases, but also revealed enlarging left hepatic lesions, an unchanged pancreatic tail mass, and a new 3.1 cm lesion in the pancreatic head (Fig. 3C).

Given the severity of illness and comorbidities, the patient transitioned to comfort-directed care and passed away on postembolization day 33.

### Case 4

An 18-year-old woman transferred care to our institution following a diagnosis of metastatic right ACC complicated by severe CS. Initial biochemical evaluation revealed markedly elevated UFC at 1022 µg/24 hours (2820.7 nmol/24 hours), DHEA-S 939 µg/dL (25.45 µmol/L), morning cortisol 25.8 µg/dL (712.0 nmol/L), ACTH <5 pg/mL (<1.1 pmol/L), testosterone 106 ng/dL, reference: <43 ng/dL (3.68 nmol, reference: <1.49 nmol/L), and androstenedione 2170 ng/dL, reference: 41 to 262 ng/dL (75.76 nmol/L, reference 1.43-9.15 nmol/L).

**Figure 4:**
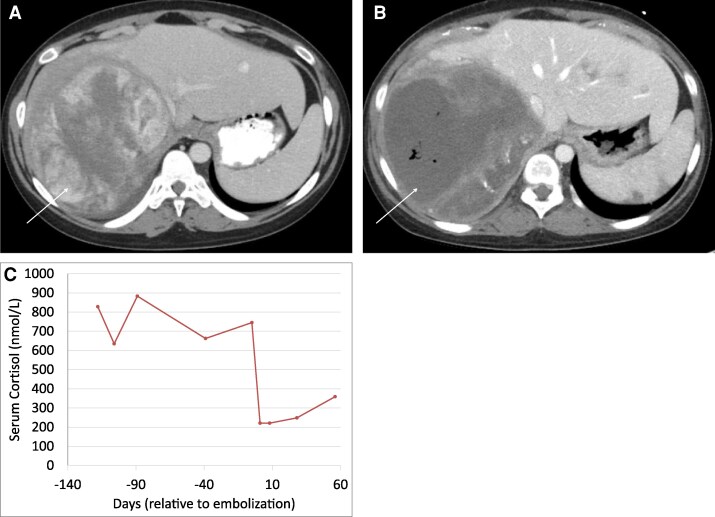
Early postembolization changes in case 4. Axial contrast-enhanced computed tomography images of a dominant heterogeneous, hyperenhancing hepatic mass in segments 7/8 prior to (A, white arrow) and 4 weeks after (B, white arrow) bland embolization. The lesion measured 12.4 × 11.2 cm pretreatment (A) and 12.0 × 11.8 cm post-treatment, with interval development of intralesional gas consistent with postembolization changes (B, white arrow). Early postembolization changes with limited biochemical response at short-term follow-up in Case 4 (C).

Cross-sectional imaging (CT and magnetic resonance imaging) demonstrated a 14 × 10 cm heterogeneous, partially calcified right adrenal mass abutting the posterior liver and displacing the right kidney inferiorly. Multiple hepatic metastases were identified, the largest measuring 12.4 × 11.2 cm in segments 7/8 (Fig. 4A), along with a 1.3 cm pulmonary nodule in the right lung base concerning for metastatic disease. 18-F FDG PET/CT confirmed metabolic activity in the adrenal mass, hepatic lesions, and pulmonary nodule. Genetic testing of 85 cancer-related genes was negative.

Initial treatment included combination chemotherapy (cisplatin, etoposide, doxorubicin), osilodrostat (maximum tolerated dose: 6 mg twice daily), and mitotane (2000 mg twice daily), along with hydrocortisone as part of a block-and-replace strategy. Due to radiographic progression, chemotherapy was transitioned to pembrolizumab. Surgical intervention was deferred due to high morbidity risk, as determined by multidisciplinary tumor board review.

Interventional radiology performed TAE targeting the right adrenal, inferior phrenic, and hepatic artery branches using 100 to 300 µm microspheres. A second order right inferior phrenic artery was also embolized using a 2 mm × 6 mm fibered coil. The procedure was completed without immediate complications.

On postembolization day 1, morning cortisol decreased to 8.3 µg/dL (229.08 nmol/L) (Fig. 4B). Osilodrostat and hydrocortisone were resumed, while mitotane was temporarily held due to elevated liver enzymes. The patient developed postembolization syndrome, characterized by fever, tachycardia, hypertension, and leukocytosis; infectious workup was negative. She was discharged on postembolization day 8.

Follow-up CT 1 month later showed stable disease in the primary adrenal tumor and hepatic metastases, with the dominant hepatic lesion measuring 12 × 11.8 cm (Fig. 4C). However, a repeat scan 2 months postembolization revealed interval enlargement of other hepatic lesions.

Currently at 3 months post embolization, the patient continues to have biochemical control with mitotane and osilodrostat and has resumed pembrolizumab.

## Discussion

The management of CS due to unresectable hepatic metastases represents a significant clinical challenge, particularly when conventional therapies such as surgical resection or medical management are ineffective or not feasible. This case series highlights the potential role of TAE and Y-90 radioembolization as minimally invasive, palliative strategies for controlling hypercortisolism and reducing tumor burden in patients with metastatic, hormonally active tumors.

Surgical resection of the primary lesion remains the first-line treatment for CS [11]. However, in patients with metastatic disease, such as ACC or ectopic ACTH-producing tumors, surgical options are often limited due to extensive tumor burden at diagnosis or poor functional status [12]. In these scenarios, where no standardized treatment sequence exists and medical therapy alone is insufficient, alternative approaches are urgently needed [5, 6, 11].

Targeted embolization techniques have been increasingly used to treat liver-dominant metastatic tumors with successful radiographic and symptomatic responses [9, 10, 13]. While their use in hormonally active tumors is not well characterized, emerging evidence (including the present series) suggests that TAE and Y-90 radioembolization may offer meaningful biochemical and clinical improvements in patients with CS due to hepatic metastases [14-16]. A summary of previously reported cases of palliative hepatic embolization for hormonally active metastatic tumors, including embolization modality and biochemical outcomes, is provided in Table 2.

**Table 2:** Clinical and radiographic features of published cases of hepatic embolization in Cushing syndrome

Author (year)	Primary tumor (size)	Primary tumor resection	Systemic therapy	Embolization technique	No. of hepatic embolizations	Biochemical response	Outcome
Doi et al (2003) [17]	Pancreatic NET (5 cm)	Surgical resection of pancreatic NET	Octreotide, metyrapone, interferon-α, adriamycin + dacarbazine streptozotocin + 5-FU	TAE	5	Biochemical response for 8 months	Biochemical and radiologic response for 8 months; subsequent tumor progression
Lin et al(2012) [18]	Pancreatic NET (3.1 cm)	Not resected (tumor burden too large)	Octreotide	TAE	1	↓ cortisol, improved serum potassium	No long-term follow-up reported
Vaduganathan et al (2015) [19]	Pancreatic NET	Not resected (tumor burden too large)	Metyrapone, octreotide	TAE	1	No significant effect on cortisol; bilateral adrenalectomy 10 days later	Died within 4 months due to postoperative complications
Zhang, et al (2020) [20]	Pancreatic NET	Not reported	Not reported	TAE	Not reported	Cortisol level returned to normal following TAE	Did not continue treatments with somatostatin analogs and lost to follow-up. Deceased 1 year later.
Watanabe et al (2022) [21]	ACC (16 cm)	Adrenalectomy	Mitotane, EDP	TAE	6	Not reported	5 of 8 liver metastases showed complete response; others <2 cm at 8 years
Blew et al (2023) [22]	Pancreatic NET (5.3 cm)	Modified Whipple procedure	Lanreotide, cabergoline, metyrapone, sunitinib	TAE	2	Persistent hypercortisolism (UFC 2479 nmol/24 hours); required additional medical therapy	↓ size/enhancement of hepatic metastases; nodal progression
Makary et al (2018) [23]	ACC (8.9 cm)	Adrenalectomy	Not reported	Y-90 (ETP)	2	↓ aldosterone; cortisol not reported	Stable disease; symptom resolution at 12 months
Pigg et al (2020) [24]	ACC (12 cm)	Adrenalectomy	Mitotane, etoposide, adriamycin, cisplatin, metyrapone	Y-90	2	Not reported	Stable hepatic disease; symptom-free at 2 years
Lu et al (2021) [25]	ACC (15 cm)	Adrenalectomy	Mitotane, ETP	Y-90	2	Adrenal crisis postprocedure; cortisol not recorded	No hepatic recurrence at 23 months
Lin et al (2022) [26]	ACC (5.1 cm)	Adrenalectomy	Mitotane, paclitaxel, cisplatin, 5-FU	Y-90	2	↓ size of hepatic metastases after Y-90; biochemical response not reported	Complete remission after RFA; disease-free at 16 months
Tamhane et al (2023) [27]	Pancreatic NET	Not resected (due to metastatic disease)	Ketoconazole, octreotide, lanreotide, capecitabine, temozolomide	Y-90	1	Decrease/stability in hepatic lesion size for most lesions, biochemical response not reported	Stable for 18 months without serious complications.

Abbreviations: 5-FU, 5-fluorouracil; ACC, adrenocortical carcinoma; EDP, etoposide, doxorubicin, and cisplatin; ETP, etoposide, mitotane, and/or platinum-based chemotherapy (context-dependent); NET, neuroendocrine tumor; RFA, radiofrequency ablation; TAE, transarterial embolization; UFC, 24-hour urinary free cortisol; Y-90, yttrium-90 radioembolization.

TAE works by selectively occluding the arterial supply to hepatic tumors, inducing ischemic necrosis and reducing both tumor volume and hormonal output [28]. In our series, 2 patients (Cases 2 and 3) with ectopic ACTH syndrome underwent TAE, resulting in rapid biochemical improvement and partial radiographic response. These findings align with prior reports demonstrating the efficacy of TAE in controlling hormone excess and tumor progression in neuroendocrine liver metastases [17-22].

Y-90 radioembolization offers an alternative approach, delivering localized radiation via microspheres to tumor vasculature. This technique allows for targeted cytotoxicity while sparing surrounding hepatic parenchyma [13, 29, 30]. In our series, the patient who underwent Y-90 embolization (Case 1) experienced significant tumor shrinkage and sustained biochemical remission. Y-90 may be particularly advantageous in cases with multifocal or bilobar liver disease, where diffuse hepatic involvement limits the utility of surgical or segmental interventions [23-27].

Both TAE and Y-90 embolization are minimally invasive, repeatable, and generally well-tolerated, with lower procedural risk compared with surgical resection [9, 31]. These techniques can be performed under local anesthesia and may serve as bridging or adjunctive therapies in patients awaiting systemic treatment or experiencing refractory hypercortisolism. While often reserved for refractory cases, the favorable safety profile and biochemical responses observed in our series suggest that transarterial therapies may also be considered earlier in the treatment course for selected patient with liver-dominant metastatic disease. Furthermore, as illustrated by Case 4, locoregional embolization can be applied not only to large hepatic metastases but also to metastatic lesions in other organs when anatomically feasible. Integration of these techniques may decrease tumor burden and also facilitate more rapid biochemical stabilization.

**Figure 5:**
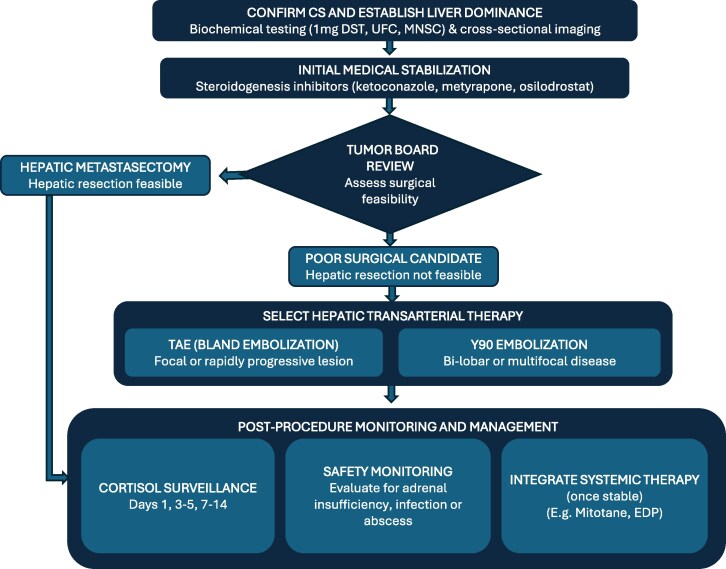
Proposed pragmatic pathway for hepatic-dominant Cushing syndrome. This algorithm illustrates the potential for transarterial therapies to serve as an additive tool for management of hepatic-dominant Cushing syndrome. While surgery and medical steroidogenesis inhibitors provide initial control, embolization techniques may also offer additional targeted tumor debulking independent of biochemical control of hypercortisolism.

Based on our experience and prior reports, we propose a pragmatic pathway: (1) confirm CS with biochemical testing; (2) establish liver dominance by cross-sectional imaging; (3) stabilize with steroidogenesis inhibitors (often block-and-replace); (4) tumor board review to assess surgical feasibility; (5) choose TAE for focal, rapidly progressive lesions or Y-90 for bilobar/multifocal disease; (6) monitor cortisol on postprocedure days 1, 3 to 5, and 7 to 14; (7) screen proactively for adrenal insufficiency and infection; (8) integrate systemic therapy once stable (Fig. 5). Despite these promising outcomes, several limitations remain. Tumor revascularization and recurrence are known risks, and hepatic dysfunction may occur, particularly in patients with underlying liver disease [14, 32]. The retrospective nature of this report, with a small number of cases, further limits the generalizability of these findings; thus, the results should be interpreted with caution. Future multicenter studies are needed to better characterize patient selection, durability of hormonal control, safety outcomes, and optimal integration of hepatic embolization with other therapies. In addition, adrenal insufficiency is also a potential concern in this patient population, underscoring the importance of screening for this condition and initiating prompt glucocorticoid therapy when indicated. Finally, the long-term impact of embolization on survival and quality of life in this population remains unclear. Future prospective studies are needed to refine patient selection, optimize embolization protocols, and evaluate the integration of embolization with systemic therapies such as chemotherapy, immunotherapy, or targeted agents. Future studies should also evaluate the long-term endocrine consequences of repeated hepatic embolization, including the incidence and recovery patterns of adrenal insufficiency, as well as the broader impact on patient-reported quality-of-life outcomes. In addition, prospective collaborative registries or multicenter consortia may help refine optimal patient selection criteria, treatment sequencing with systemic therapies, and standardized monitoring protocols to improve safety and durability of biochemical control.

In conclusion, hepatic TAE and Y-90 radioembolization are effective, minimally invasive options for palliating CS due to liver-dominant metastatic disease. These techniques may serve as additive tools within a multimodal treatment strategy in selected patients with liver-dominant metastatic disease. These liver-directed interventions can provide meaningful biochemical control and tumor reduction when conventional treatments are insufficient. Although our findings primarily pertain to hepatic metastases, targeted embolization of extra-hepatic lesions may also offer therapeutic benefit in carefully selected cases.

## Data Availability

Some or all datasets generated during and/or analyzed during the current study are not publicly available but are available from the corresponding author on reasonable request.
